# Wetland Restoration with Hydrophytes: A Review

**DOI:** 10.3390/plants10061035

**Published:** 2021-05-21

**Authors:** Maria A. Rodrigo

**Affiliations:** Integrative Ecology Group, Cavanilles Institute for Biodiversity and Evolutionary Biology, University of Valencia, Catedrático José Beltrán 2, 46980 Paterna, Valencia, Spain; maria.a.rodrigo@uv.es

**Keywords:** revegetation, submerged macrophytes, floating macrophytes, aquatic phanerogams, charophytes, seeding, planting, transplanting, sediment transfer, natural wetlands, constructed wetlands

## Abstract

Restoration cases with hydrophytes (those which develop all their vital functions inside the water or very close to the water surface, e.g., flowering) are less abundant compared to those using emergent plants. Here, I synthesize the latest knowledge in wetland restoration based on revegetation with hydrophytes and stress common challenges and potential solutions. The review mainly focusses on natural wetlands but also includes information about naturalized constructed wetlands, which nowadays are being used not only to improve water quality but also to increase biodiversity. Available publications, peer-reviewed and any public domain, from the last 20 years, were reviewed. Several countries developed pilot case-studies and field-scale projects with more or less success, the large-scale ones being less frequent. Using floating species is less generalized than submerged species. Sediment transfer is more adequate for temporary wetlands. Hydrophyte revegetation as a restoration tool could be improved by selecting suitable wetlands, increasing focus on species biology and ecology, choosing the suitable propagation and revegetation techniques (seeding, planting). The clear negative factors which prevent the revegetation success (herbivory, microalgae, filamentous green algae, water and sediment composition) have to be considered. Policy-making and wetland restoration practices must more effectively integrate the information already known, particularly under future climatic scenarios.

## 1. Introduction

Several types of water bodies are included in the term “wetland”. For example, Kettenring and Tarsa [1] cited inland freshwater and saline wetlands (emergent wetlands, sedge meadows, wet prairies, fens, vascular plants in bogs, and temporary or seasonal wetlands, such as vernal pools and mudflats), coastal marshes (salt, brackish and freshwater tidal), seagrass meadows, and forested wetlands (riparian, floodplain, bottomland hardwood, mangroves, etc.). The same authors [1] and others [2,3,4,5] have stressed the essential ecosystem functions and services that wetlands provide in the world: wetlands improve water quality for downstream waters but also support biodiversity for conservation, they mitigate flooding, drought and climate change effects and combat sea-level rise, protecting coastlines, but also provide habitat for recreation, culture and other activities. In spite of this, human societies have negatively impacted wetlands throughout history, with the result of a loss of more than half of the wetlands globally. This represents high impacts and risks to wildlife, and human economies and health [1]. Therefore, the restoration of this type of habitats is a must for the welfare of humanity. In March 2019, the Decade of Ecosystem Restoration 2021–2030 was declared the United Nations [6] and thus, integral wetland restoration must be considered within these priorities and efforts.

One of the active strategies for aquatic ecosystem restoration has been traditionally seedling or planting macrophytes [7,8,9]. However, the majority of these study cases focusses on emergent aquatic plants, such as *Typha* spp., *Juncus* spp., *Phragmites australis*, etc. [10,11,12]. Restoration cases with hydrophytes, understanding them as the aquatic “plants” in a strict sense (that is, those which develop all their vital functions inside the water or very close to the water surface as the case of flowering; thus, they live submerged or floating in the water) are less abundant. Among other reasons, it is because working with hydrophytes is much more challenging compared to emergent plants. Moreover, much of this information is broadly scattered throughout the peer-reviewed and grey literature. Hence, there are no synthetic comprehensive reviews for restoration of wetlands based on hydrophytes. Here, I synthesize the latest knowledge about restoration of wetlands based on revegetation using hydrophytes. I also stress the common challenges and propose potential solutions. Within hydrophytes, phanerogams, macroalgae and aquatic pteridophytes and bryophytes (mainly mosses and liverworts) can be considered. However, this review is restricted to submerged and floating phanerogams in continental wetlands, macroalgae, such as charophytes, or seagrasses for coastal wetlands. The conservation consensus is clear: “the protection of intact undisturbed environments is the only real solution to conservation of natural communities”. However, what to do with those wetlands already affected? Revegetation is not the perfect solution for the conservation of wetlands. This is because many times it is not able to perpetuate species at risk, nor maintaining complex natural communities, but less is nothing. Therefore, this review mainly focusses on natural wetlands but also includes some information about more or less naturalized constructed wetlands, which nowadays are being used not only to improve water quality [13] but also to increase biodiversity and recover other ecosystem services such as carbon sequestration [14,15,16,17]. Available publications from the last 20 years (both peer-reviewed and any public domain) have been reviewed (although I might have missed some cases). Personal expertise is also provided. I address two scales of interventions: (i) outdoor experimental approaches and (ii) large-scale actions in the field. I focus on issues related to the different approaches used (seedling, planting, transferring sediment, etc.). I also discuss the most common hydrophyte species used in restoration, the factors affecting revegetation and stress the challenges to evaluate the success of revegetation.

## 2. Natural and Constructed Wetlands

The ideal situation would be, of course, the preservation of natural wetlands whenever possible and that the reconstruction should be considered only as a last resort [18]. This is sometimes not possible because natural wetlands have disappeared or are severely degraded, and constructed wetlands (CWs) are implemented. CWs are artificial wetlands which have been designed to intercept wastewater and remove a broad range of pollutants before being discharged into natural water bodies [19]. Surface-flow CWs are similar to natural marshes, usually located in shallow channels and basins through which the water flows slowly above the substrate [20]. CWs mimic natural wetland ecosystems and combine physical, chemical, and biological processes to purify the water in more-controlled and efficient ways [19]. On the other hand, the aim of wetland restoration is to restore lost biodiversity and to provide ecosystem services (i.e. flood-peak reduction, water-quality improvement using phytoremediation) [9]. A successful restoration project may need to incorporate different types of wet environments (see [9]), with the combination of areas for phytoremediation with areas of low nutrient content. In the last two decades, there has been an increasing trend to implement CWs in protected areas, such as national or natural parks in all continents (e.g., in Europa: Italy [21,22], Spain [16,23], Poland [24]; America [25,26]; Asia [27]; Africa [28]; Oceania [29], etc.). Some authors stress that wetland restoration must be prioritized over the creation of artificial wetlands, because, even when intended for conservation, they may not provide an adequate replacement of, for example, waterbird-supported functions [30]. However, other authors indicate that the biodiversity of constructed wetlands for wastewater treatment can be enhanced through proper design and management [31]. Moreover, CWs may serve as experimental pilot areas where treatments and procedures for revegetation to be further applied in wider natural but degraded wetlands can be tested [16].

## 3. Leader Countries in Wetland Revegetation with Hydrophytes

**Figure 1:**
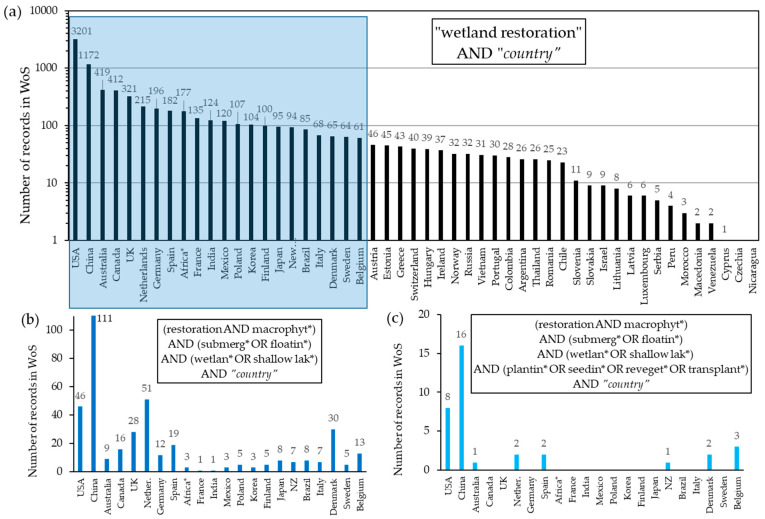
(**a**) The number of records obtained in the Web of Science (WoS) search for wetland restoration by countries (*except for the African continent) (notice the logarithmic scale). (**b**) The number of records in the WoS with the keywords: (restoration AND macrophyt*) AND (submerg* OR floatin*) AND (wetlan* OR shallow lak*) AND “*country*”, for the countries that showed more than 60 records in the previous search (graph (**a**)). (**c**) Number of records for the countries of graph (**b**) now with the keywords: (restoration AND macrophyt*) AND (submerg* OR floatin*) AND (wetlan* OR shallow lak*) AND (plantin* OR seedin* OR reveget* OR transplant*) AND “*country*”. Searches made in March 2021.

Since almost all countries in the world have their wetlands affected by pollution and many other problems, a large part of them has attempted to restore wetlands. However, in countries, where there are many large lakes, restoration has focused on them. In countries, where the scarcity of large continental water bodies is the normal situation, such as countries in the Mediterranean, with a semiarid climate, wetlands take a special relevance. The United States of America (USA) is the first country in the ranking of records obtained in a search in the “Web of Science” (WoS) about “wetland restoration” by world countries (more than 3000 records), followed by China (more than 1170 records) (Figure 1a). Australia, Canada, United Kingdom, and The Netherlands showed between 419 and 215 records. Germany, Spain, Africa (mostly South Africa), France, India, Mexico, Poland, Korea and Finland yielded between 196 and 100 records. Japan, New Zealand, Brazil, Italy, Denmark, Sweden and Belgium showed between 95 and 61 records. The rest of the countries showed less than 60 records.

Firstly, among the 22 countries with more than 60 records, the highest number of records in the search in the WoS including submerged or floating macrophytes (using the string (restoration AND macrophyt*) AND (submerg* OR floatin*) AND (wetlan* OR shallow lak*) AND “*country*”), was for China with 111 records (Figure 1b), followed by The Netherlands, USA and Denmark. UK, Spain and Canada showed between 28 and 16 records. The rest of the countries produced less than 13 records. Secondly, focusing on revegetation approaches and adding to the above string “AND (plantin* OR seedin* OR reveget* OR transplant*)”, the number of records was substantially reduced (Figure 1c), but China was again the country with the highest number of records (16), followed by the United States (8).

As seen above, both in Europe and the United States, but also in China, there has been a large tradition of aquatic ecosystems restoration [32,33,34,35]. For example, important native seed banking initiatives have been developed to improve the access to genetically diverse native wetland seeds for research, conservation and restoration (European Native Seed Conservation Network (ENSCONET) and Seeds of Success (SOS)) (see references in [1]). China, with 68.5 million hectares of wetlands (36.2 and 32.3 million ha natural and constructed wetlands, respectively [27]), is one of the countries where many attempts at submerged macrophyte restoration and bioremediation have been made since the 1980s, despite the unfavorable results in some of the cases (references in [36]; reviewed in [37]). For example, from 2002 to 2006, nearly 60 programs have been developed to restore wetlands in this Asian country [38]. Their main results suggest that, combined with other technics (e.g., addition of filter-feeding aquatic animals in the proper biomass), submerged macrophyte restoration in wetlands might have a high success rate. The restoration or rehabilitation of wetlands around rivers and lakes has also been growing rapidly since the early 1990s in Japan including activities to recover lost or degraded vegetation and plant diversity [39]. In New Zealand, the most common action undertaken for restoration of wetlands is revegetation, involving removal of introduced weeds, and then the planting of native species appropriate to the habitat conditions and region [40].

## 4. The Scale of Revegetation

Some studies dealt with indoor aquaria or smaller outdoor mesocosm with the prospect of future wetland or shallow lake restoration program centered on hydrophyte replacement (see, for example, Ciurli et al. in Italy [41] or Fontanarrosa et al. in Argentina [42]). However, for successful revegetation, research using experimental on-site wetland mesocosms should be planned before starting larger-scale initiatives. Thus, it is possible to reduce the failure rate by improving the knowledge of the species ecological requirements, the introduction method, the selection of ecologically suitable translocation sites and the choice of source material [43]. Moreover, as revegetation represents a large proportion of the costs associated with restoration, developing cost-effective new planting methods would reduce the costs of large-scale restoration [44].

Table 1 shows some of the experiments which have been conducted related to revegetation with hydrophytes. Qiu et al. [45] performed in situ enclosure experiments in three parts of a eutrophic shallow lake in China with different trophic status, introducing both floating-leaved and submerged hydrophytes. All the introduced species grew well. The authors reported a monthly mean macrophyte biomass increase of 329 gWW/m^2^. A large-scale experiment was conducted in the Danish shallow Lake Engelsholm, where three species were planted in three 25-m^2^ exclosures with densities of 4–10 ramets/m^2^ (with no roots) and 25 cm in length for *Stuckenia pectinata* and 40–50 cm in length for the other two *Potamogeton* species [46]. After two years following transplantation, the plant density development increased six-fold. Hilt et al. [47] described how Rott, in 2002, planted 200 m^2^ of “macrophyte islands” with *Myriophyllum* spp. and *Chara contraria* in a 25-ha shallow lake in Southern Germany. One year later, the hydrophytes had already colonized 5.3 ha, which represented a monthly coverage increase rate of 0.4 ha. Ye et al. [48] performed an ecological restoration demonstration project in the shallow lake Taihu planting a total density of 105 plants/m^2^ of four species of submerged hydrophytes in containers. After one year, *H. verticillata* dominated the composition of the communities, with only a few *P. malaianus, V. spiralis* and *N. marina* remaining, owing to the competitive exclusion from *H. verticillata*. Moore et al. [49] demonstrated with exclosure experiments that *Vallisneria americana* can be successfully restored in tidal freshwater areas of the Chesapeake Bay that were unvegetated for 60 years. These authors concluded that whole shoot transplants resulted in the most rapid cover, whereas direct dispersal of individual seeds or intact seed pods were also effective, but the recovery was slower. When protected from herbivory, approximately 3 years of growth were required for the transplants to reach 100% bottom cover at maximum densities of 100–150 shoots/m^2^. Rodrigo et al. [16] set up 54 1 × 1 m-exclosures in two shallow lagoons within a newly created constructed wetland in Spain that were planted with cultures of two species of higher plants and two charophyte species (16 cultures of three specimens each/m^2^). The higher plants developed better than the charophytes, but always when protected from biotic factors. Gao et al. [50] performed outdoor mesocosm experiments with four species of submerged macrophytes, planted at a density of 300 gWW/m^2^ in the Gonghu Bay of Lake Taihu. They concluded that *H. verticillata* and *P. malaianus* are suitable submerged macrophyte species for restoration of eutrophicated shallow lakes. The relative growth rate of *H. verticillata* was maximum (0.03), and around 0.01 for the other three species. In Sweden, Nilsson et al. [51] planted *Elodea canadensis*, *Myriophyllum alterniflorum* and *Ceratophyllum demersum* in newly semi-natural constructed wetlands to intercept nitrogen from surface waters in an agricultural landscape and followed them for 12 years. Nitrogen removal increased with the ecosystem age, and the dominant submerged species was *Potamogeton natans*, which colonized naturally. Schad et al. [52] planted founder colonies of *Heteranthera dubia* and *Potamogeton nodosus* in a series of constructed floodway wetlands in the USA to analyze the influence of varying construction completion dates, water sources and ecosystem management stage on macrophyte development and its relationship with macroinvertebrate assemblages.

For macrophytes to maintain a clear water state, a minimum coverage of shallow water bodies is required [53]. As a rule of thumb, 30% coverage has been used as a minimum threshold, which is in the range of 10–40% reported by some authors, but lower than 50% indicated by others. In warm shallow lakes in tropical and subtropical regions, even a higher hydrophyte coverage may be needed as the grazing of zooplankton on phytoplankton is low due to high fish predation (see references in [53]). In Mediterranean regions, where high temperatures are reached in summer, a larger coverage would be necessary to outcompete the growth of phytoplankton and filamentous algae. Consequently, large-scale restoration efforts should be faced because they could potentially be more successful than smaller ones since large submerged aquatic vegetation beds are thought to be more stable and resilient to stress than small beds [54]. However, getting such high coverages requires a tremendous effort (high costs for material, installation and maintenance and solving difficulties, such as filamentous algal growth or high herbivory pressures, and interference with recreational use). Therefore, large-scale plantings of hydrophytes have not often been performed in wetlands, although there are some cases. Table 2 gathers study cases of hydrophyte revegetation at larger scales (from 0.4 ha or more). One of the most emblematic cases of a large-scale approach to submerged aquatic vegetation restoration is the Chesapeake Bay, the first estuary in the USA to apply an integrated watershed approach for restoration (13.4 ha/year were revegetated in 2003–2008 [44]). In New Zealand, Dugdale et al. [55] planted 1 ha of a shallow lake with charophytes protected from fish and it was successful in allowing founder colonies of charophytes to establish and expand (≥75% cover within one year). At the Mediterranean, Sebastián et al. [56] restored two wetland areas in Spain by planting *Ceratophyllum demersum*, *Myriophyllum verticillatum*, *M. spicatum* and the floating-leaved species *Nymphaea alba* as well as two shallow lagoons at the end of a constructed wetland [16]. In China, Chen et al. [57] bordered an area of 10 ha of the littoral zone of lake Wuli (a bay of Taihu lake) with a waterproof fabric and planted four species of submerged macrophytes, three species of floating-leaved macrophytes and one species of a free-floating plant. The coverages of floating-leaved macrophytes, submerged macrophytes and free-floating macrophytes inside the enclosure were up to 9.7%, 8.1% and 2.9%, respectively, one month after plantation. One year later, the coverage area of aquatic macrophytes (including emergent species) expanded and increased to about 45.7%. Clarkson and Peters [40] revegetated New Zealand wetlands by planting *Potamogeton cheesemanii*, *Myriophyllum propinquum* and *Lemna minor*. Dick et al. [58] planted six species of submerged hydrophytes, one species of charophyte and four species of floating-leaved species in a chain of wetlands in the USA. Plant establishment continued with supplemental plantings 4–10 years later. Yu et al. [59] found that restoration by transplantation of six species of submerged macrophytes (at a density of 30–70 adult plants/m^2^) after fish removal had major positive effects on water quality variables in three shallow lakes of China (an isolated 5-ha bay of shallow lake Taihu, lake Qinhu (8 ha) and South lake (0.4 ha)). Theÿsmeÿer et al. [60] planned the recovery of submerged and floating-leave species in the Canadian marsh area of the Great Lakes. More recently, Liu et al. [61] reported the successful restoration of a tropical shallow eutrophic lake combining fish removal with transplantation of submerged macrophytes. *Vallisneria denseserrulata* was planted at a density of 10–15 shoots/m^2^ and *Hydrilla verticillata* of 20–30 shoots/m^2^.

Revegetation is essential, but whole-ecosystem, long-term interventions including most if not all ecosystem processes are desirable to be sure that the restoration result is the expected [62]. Furthermore, for large-scale hydrophyte restoration, the efforts should be in the framework of coordinated interagency programs, to develop, evaluate, and refine the suitable protocols and procedures. Maybe this is an issue not so easy to achieve. Guidelines should be published to help managers aiming to restore wetlands and shallow lakes, and critically assess and predict the potential development of submerged vegetation, considering the complexity of factors and interrelations determining their occurrence, abundance and diversity. In very few countries (such as the USA in 2002 [63] and Germany in 2006 [47]) such guidelines were published.

## 5. Procedure Approaches in Revegetation with Hydrophytes

### 5.1. Seeding

The need to seed in wetland restoration has been widely and recently reviewed by Kettenring and Tarsa [1]. For wetland restoration, the approaches based on strategies which utilize seeds to recover in totality the composition, structure and functions of the submerged vegetation, which support their ecosystem services, are an initial phase, particularly to treat larger areas. This is because the seed-based strategies are less expensive, in spite of the elevated prices of native seeds, and more logistically achievable than revegetation techniques such as installing mats, transplanting rhizomes or planting plugs (see [1] and references therein). However, frequently a deficiency of submerged macrophyte propagules is faced. This is why, on some occasions and for certain plants, in vitro propagation protocols have been developed [38].

Moreover, seed mortality uses to be high, therefore the final result of seeding is not easy to predict ([1] and references therein, for example, [43,64]). Since the seed survival is low, both seed and seedling stages represent an important bottleneck for the demography of plant populations [65]. Hence, ecological, evolutionary and genetic principles should be considered in effective approaches based on seeding. Economic, logistic and social limitations are also important [1]. Moreover, to have successful revegetation results, those environmental factors which can result limiting have to be handled, together with the application of proper management actions [1].

In many wetlands, recalcitrant seeds occur [1]. These species require storage either under high humidity or submerged in water. It has been shown [1] that seeds kept under submerged storage conditions show higher seed longevity with aeration (this is the case of *Zostera marina* and *Potamogeton perfoliatus*), or under cool temperatures (e.g., *Z. marina*, *P. perfoliatus* and *Ruppia maritima*) or high salinity (e.g., *Z. marina*, *R. maritima*; ([1] and references therein, [66,67,68])). Some species of submerged vegetation can also be stored under low humidity/low moisture for some temperatures. This is the case of the Hydrocharitaceae and Potamogetonaceae families [69,70], and thus may not be recalcitrant contrary to general predictions in aquatic plants. In the case of charophytes, it has been described how, in some situations, their seedlings do not progress in their original habitats, although restoration practices have been performed to recover the best physical and chemical conditions for their growth [70]. These macroalgae produce extremely small propagules (oospores), and working with them is not an easy task. Recently, a protocol consisting of microencapsulating these oospores using sodium alginate has been published [71], and it is presented as a promising method for preserving charophyte oospores to support both laboratory and field experiments. The author proposes this procedure to greatly facilitate the conduct of both in situ and ex situ conditions’ studies and experiments.

The seed sowing has to be done at the earliest but within the temperature optimal range of each species. Special care has to be taken to avoid light inhibition at the surface of the sediment due to the development of the plant canopy [1]. Regarding recommendations for rates of seed sowing, the data are scarce and are different depending on the type of wetland [1]. For example, Broome et al. [72] sowed 100 pure live seeds (PLS)/m^2^ in tidal salt marshes. Busch et al. [73] used 37 PLS/m^2^ of *Zostera marina*. In the large Chesapeake Bay restoration (41 ha), *Z. marina* seeding densities of 11 to 49 seeds/m^2^ were used [44]. Current restoration techniques for seed sowing introduce the seeds manually or using machines and they have been designed to overcome limitations in the dispersal; however, they do not necessarily mimic the dispersal mechanisms occurring in nature [1], which comprise water, wind, animals (particularly waterbirds) and gravity. One approach for keeping seeds in place is sowing seeds in burlap bags made with natural fibers. This has also been used with *Z. marina,* and it improves the recruitment outcomes in seagrass meadow restoration with high wave action [74]. To avoid the low seedling establishment rates (<10%) and seed loss through herbivory on seeds, mechanical devices for planting *Z. marina* seeds slightly beneath the sediment surface have been developed with improved seedling establishment rates [67,75,76]. To vegetate large wetland areas, aerial seeding might be both economical and practically feasible [77]. With this, the restoration efforts could be accelerated without using expensive hand-planting methods of vegetative clones (see below), but the unavailability of large quantities of viable seed is one of the major hindrances [77].

**Figure 2:**
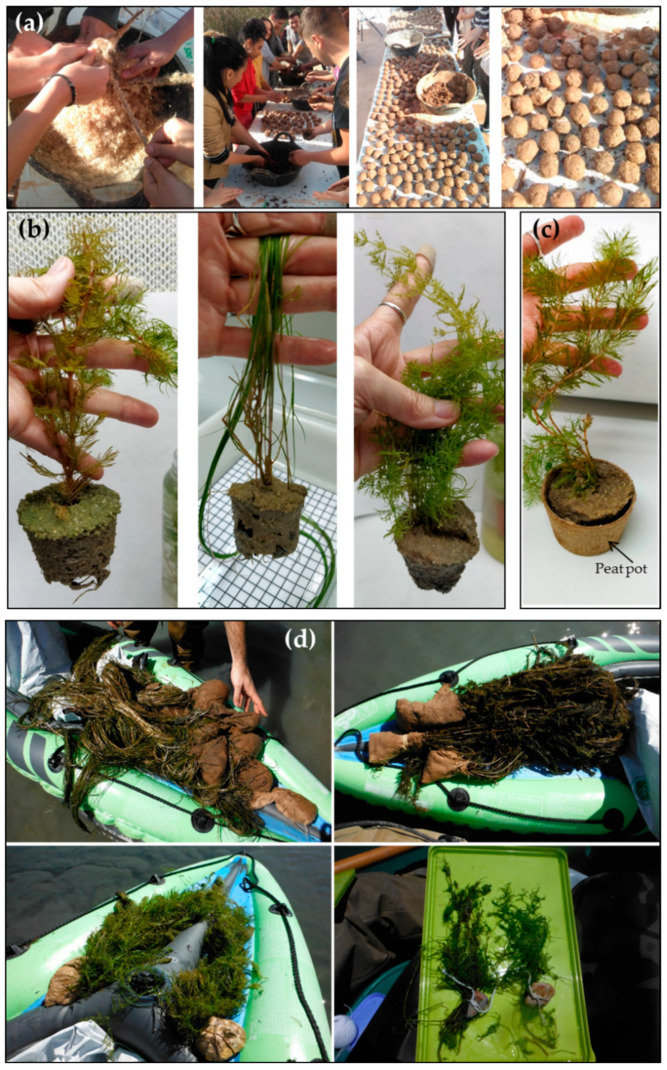
(**a**) Seed bombs with *Typha* seeds prepared by students with the supervision of the managers of Tancat de la Pipa wetland (Albufera de València Natural Park, Spain; photographs: Lourdes Ribera); (**b**) examples of *M. spicatum*, *S. pectinata* and the charophyte *Chara vulgaris* ready to be planted in the field without any kind of holder; (**c**) example of *M. spicatum* with the root-sediment system in a peat pot ready to be planted in the field; (**d**) fragments of hydrophytes with a stone to serve as “anchor” to be thrown inside enclosures (see Figure 2c) (*S. pectinata*, *M. spicatum*, *C. submersum* and *C. vulgaris*).

Seed pellets (also known as pods, seed balls and seed bombs), which are an aggregation of clay, soil, water, and multiple seeds, have been used in terrestrial restoration [78,79] and have been successfully used with emergent plants in wetlands (*Typha* seeds; Moreno L., pers. comm., Figure 2a). Thus, seed pellets are potentially a user-friendly way to establish wetland plants because they can be launched into the air in cases of hard-to-reach locations. No information has been found for the use of seed pellets in the case of hydrophytes. Therefore, this is a potential study field in the restoration of wetlands with hydrophytes, but it needs further research and testing. Moreover, making seed bombs has been a popular activity at garden centers, family events and student visits (Figure 2a), and it is potentially a viable method for engaging and educating the society, at the same time, it helps distribute native seeds across larger areas. 

Mechanical planters designed for planting whole seagrass plants and sods have been developed in both the United States and Australia [44]. In spite of their limitations in the operating procedures (e.g., depth limits, donor bed proximity, weather, need for SCUBA divers), they hold the potential for rapidly and cost-effectively planting larger areas of submerged aquatic vegetation than would be possible through manual means. For wetlands, SCUBA divers are not necessary, due to their shallowness, but large mechanical planters can have a great impact on the wetland fauna since planting should be done in spring when waterfowl is also breeding. 

### 5.2. Planting: Translocations and Production of Hydrophyte Cultures

Translocations are among the techniques used in wetland restoration and plant conservation [9,80] (i.e., the human-mediated movement of living organisms for restoration and/or conservation benefit from one area, with release in another). If hydrophytes are not taken from existing areas, and to minimize potential impacts to wild populations, they must be cultivated previously to be planted. Hydrophytes can be cultured in aquaculture systems (as described in Tanner and Parham [81]). Zhou et al. [38] described how a 15 m^2^ tissue culture room together with a 50 m^2^ acclimation pond can produce 125,000 high-quality seedlings for *Myriophyllum spicatum* and *Potamogeton crispus* in 7 weeks, which could vegetate more than 2 ha sediments in shallow lake recovery programs, with the common density adopted in East China. Rodrigo et al. (unpublished results) prepared 2000 cultures of *M. spicatum*, *Stuckenia pectinata* and several species of charophytes in a 9-m^2^ culture room in 8 weeks, ready to be planted in a Mediterranean wetland. Regarding other cultivation times, *Zostera marina* plants large enough for planting can be grown from seeds within 70–100 days under controlled conditions [81]. Rodrigo and Carabal [17] reported lengths of 80 cm for *Stuckenia pectinata* in one month planted from 10-cm cuts and cultured in an acclimated room; *M. spicatum* and *Chara vulgaris* grew up to 25 cm in one month starting from 5-cm apical parts [17]. Riis et al. [7] indicated that submerged plant shoots of 20–25 cm are adequate to be planted. Moreover, they recommend using shoots with an apical tip, since they have been previously shown to regenerate better than shoots without. For the case of constructed wetlands, the use of innovative tissue culture technologies allows isolation of plant clonal lines of single seed phenotype origin that can be screened for particular tolerances, such as cold temperature, high nitrate removal rates, etc. [82]. The sediment used for preparing the cultures should preferably be from the local site but if it is impossible to get enough top sediment from the selected wetland, the volume can be augmented with a mixture of commercial sand and sediment [16].

Planting of submerged hydrophytes can be done directly with the root-sediment “ball” (Figure 2b) or by using biodegradable holders. Rotting and non-rotting substrates (e.g., nets) that keep planted macrophytes on the waterbody bottom have been used in Germany [47]. Likewise, other types of substrate have been utilized on some occasions: wood cages [56] and peat pots [16] (Figure 2c) in Spanish wetlands, trays in rivers [7]. However, peat is an example of a non-renewable resource and its extraction could contribute to the degradation of wetland ecosystems. For this reason, a biodegradable material which, at the time is a waste, as is the case of rice straw in particular areas, could represent a good option to make holders for planting. For example, in the Albufera de València Natural Park (València, Spain), there are 15,000 ha of rice fields, which produce 80,000 tons of rice straw per year [83]. The elimination of this “waste” is a problem in that area. Part of this material could be used in the fabrication of biodegradable pots, after appropriate research, for restoration tasks. Rice straw substrates have already been used in floating beds planted with macrophytes for treating wastewater. This substrate enhanced bio-remediation efficiency and macrophyte growth in comparison to the inert palygorskite ceramsite which hindered the bio-remediation process [84]. On some occasions, planting has been performed using just a stone adhered to the fragments of plants (Figure 2d). This has been successfully used even with charophytes (Rodrigo et al., unpublished results; Figure 2d). Regarding charophytes, Blindow and Carlsson [85] (this issue) describe methods for oospore germination, cultivation and plantation of charophytes depending on the type of charophyte species (k-strategists vs. r-strategists) to support the existence of threatened species in Sweden. This knowledge might be used in a wider framework for restoration purposes.

Hydrophyte planting can be done by hand [16,59], etc., or mechanically [44]. Planting by hand is rather work-intensive and might ideally involve volunteers. Planting of hydrophytes is not a complicated task, and volunteers (e.g., undergraduate and master students, members of NGOs, etc.), following wetland managers and researchers’ instructions, can do a great job. Furthermore, it may be worth doing to increase outreach and involve the local population in recovery efforts.

**Figure 3:**
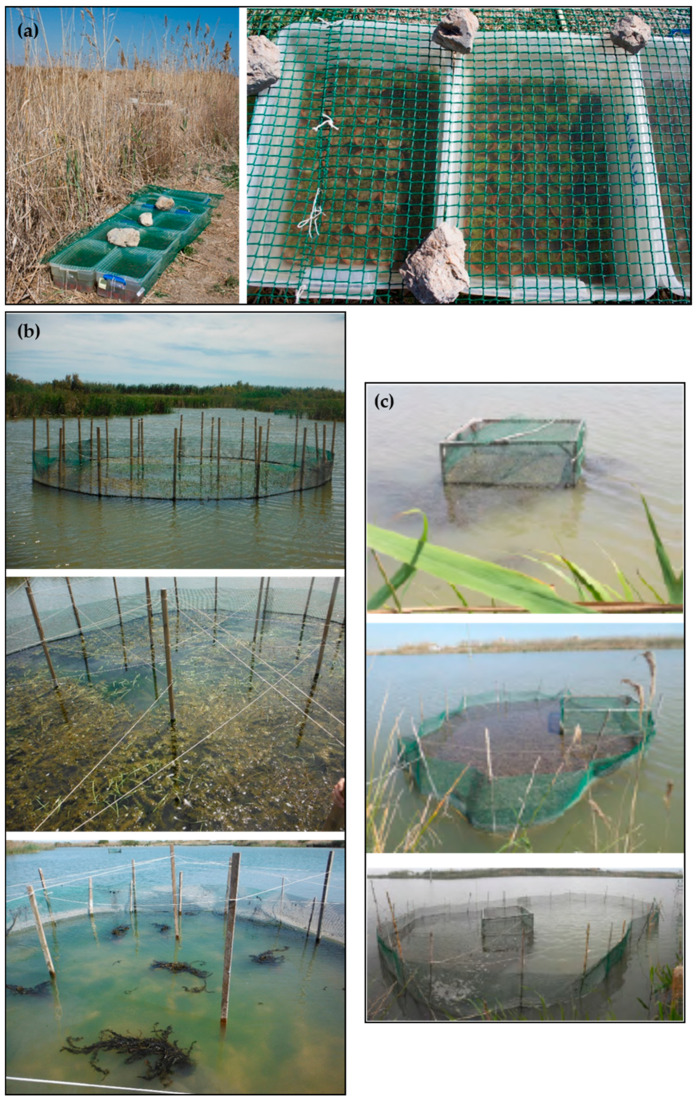
(**a**) Tanks of hydrophyte cultures produced indoors, now acclimatizing in the field before being planted in Tancat de la Pipa wetland (Albufera de València Natural Park, Spain; photographs: Ximo Fernández). (**b**) Exclosures to plant hydrophytes using the method shown in Figure 2d; the exclosure is almost 100% covered with the grown submerged macrophytes (the thin ropes in the upper part try to avoid grazing by herbivorous birds). (**c**) Exclosures to plant hydrophytes that are being enlarged successively to get wider coverages (photographs taken from [86]).

Plants cultivated indoors might be subjected to significant differences in ecological conditions between the cultivation site and recipient site (the larger the differences, the greater the probable unwilling impact on the introduced plants’ survival and fitness). Therefore, to reduce environmentally mediated shocks, acclimatization techniques (hardening) must be adopted before locating the cultures to the recipient site. One method consists of placing the plant cultures in acclimation ponds close to the target wetland if available, or in tanks located in the wetland to be revegetated, for gradual acclimatization to external temperatures, decreased or increased shading, etc. (Figure 3a).

Once the restoration has been performed by planting, and if most of the plants have been produced by vegetative reproduction, seed pellets could be thrown near the hydrophyte stands to increase the genetic variability.

### 5.3. Sediment Transfer/Transplantation

Effective transplanting of wetland soil from small remnant wetlands in other areas of shallow marshes has been performed on several occasions [87,88,89,90]. However, most of the studies refer to wet meadows, *Sphagnum*-dominated peatlands, etc., and the study cases with sediment transfer involving submerged or floating species are few (Table 3). Brown and Bedford [88] observed little plant establishment at water depths greater than 45 cm, suggesting that transplantation of wetland soil should be concentrated in shallower zones; therefore, this method seems not to be the most adequate for submerged macrophytes that grow deeper. On the other hand, transfer of bulk soil has shown promising results in temporary wetlands, indicating that soil transfer may enhance the success of wetland restoration projects compared to natural colonization [91,92]. This technique has been described as the most efficient method for transferring large numbers of short-life plant species in temporary wetland because they can generate large quantities of seeds rapidly forming large seed banks [92,93]. Muller et al. [92], in a temporary wetland restoration after rice cultivation in France (Table 3), found that soil transfer not only enhanced the target species introduction but also significantly reduced the establishment of undesired species emerging from the seed bank and from the surroundings, such as ricefield weeds (mostly exotic species introduced by rice cultivation). The pilot project of revegetation of lakeshore vegetation launched at Lake Kasumigaura in Japan by transferring lake bottom sediment achieved the recovery of 12 native submerged plants during the first year of restoration previously disappeared [91,94] (Table 3). Soil transfers also have the advantages of biotic interactions preservation by transferring soil microorganisms, important in structuring plant community and improving substrate conditions, and the transfer of zooplankton and macroinvertebrate egg bank [92]. However, nature protection aspects and the potential risk of transferring pollutants, or undesired species (such as fish or other animals’ parasites, pathogens, etc.) must be considered.

## 6. Selection of Species. Most Commonly Used Species

Different authors have indicated that the selection of hydrophytes for revegetation should be made based on (i) the type of wetlands considered, (ii) the former vegetation in the wetland and the species typically occurring in that type of water and region, (iii) the potential uses of the wetland (natural vs. constructed wetland), (iv) the suitability of the selected species for seeding and/or planting, (v) the habitat preferences of the selected species and (vi) the potential origin or source of the plants (or seeds). Moreover, the tools for the restoration of aquatic plant communities should consider the complex interactions between abiotic factors and aquatic plant requirements; otherwise, the objective of restoring such communities may be difficult to reach [95]. Recently, it has been stated that the efforts to build and maintain the resilience of an ecosystem after restoration by revegetation should be trait-based rather than merely focusing on vegetation abundance [96]. In addition, Song et al. [97] showed that the macrophyte effects on water quality vary by growth forms and that the growth forms which positively affect the water quality differ between the (sub)tropical and temperate areas. Dalla Vecchia et al. [98] stressed that root traits may explain important plant functions and need further research. Su et al. [96] suggested that plant height was one of the mechanisms underlying the positive feedbacks on water quality. Submerged plant species of taller-growing “rank”, such as *M. spicatum* and *Stuckenia pectinata* have been suggested to be introduced initially in coastal eutrophic wetlands [17]. Choosing between r-selected and k-selected plants is also crucial. For example, Qiu et al. [45] attributed the failure of recovery of *P. maackianus,* a k-selected species, when it was used as initial species, to its poorly developed rhizome, weak regeneration capacity and relatively small seed bank. Pioneering species should have been used first for restoration and *P. maachianus* and other perennial plants could be re-introduced later to increase the biodiversity [45].

Hilt et al. [47] recommended the following submerged macrophytes species for potential successful use for artificial colonization in eutrophic shallow lakes in Germany: *Ceratophyllum demersum*, *Chara contraria*, *C. globularis*, *Nitella mucronata*, *Eleocharis acicularis*, *Myriophyllum spicatum*, *M. verticillatum*, *Najas marina*, *Potamogeton alpinus*, *P. berchtoldii*, *P. crispus*, *P. friesii*, *P. obtusifolius*, *P. pusillus*, *P. perfoliatus*, *Stuckenia pectinata*, *Ranunculus* subg. *Batrachium, Ranunculus trichophyllus* (only in alkaline lakes), *Zannichellia palustris* ssp. *palustris. H. verticillata* and *P. malaianus* have been described as suitable submerged macrophyte species for restoration of eutrophicated lakes and wetlands [50] when combined with filter-feeding aquatic animals. *Myriophyllum verticillatum*, *Potamogeton perfoliatus* and *Najas minor* yielded quite similar results when nutrient removal efficiencies were analyzed, although they were higher for *N. minor* and Zhou et al. [99] pointed at *N. minor* to be a promising plant for water purification. On some occasions, facilitation has been the proposed mechanism that may enhance the colonization of several submerged hydrophytes planted at the same time [100]. Thus, Dai et al. [101] proposed using the combination of *C. demersum* and *M. verticillatum* as the best choice for ecological restoration of eutrophic water bodies. The charophyte *Chara vulgaris* has also been used for replanting in eutrophic wetlands due to its high-nitrate concentrations tolerance and because it is a r-strategist that produces large amounts of oospores [102]. 

**Figure 4:**
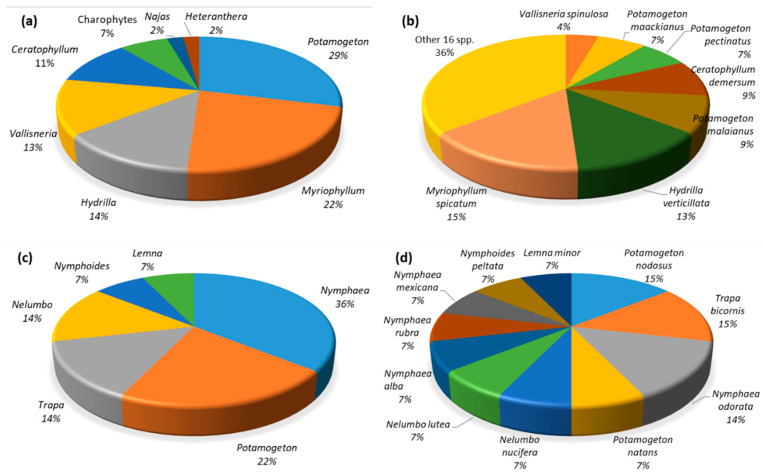
(**a**) Distribution of the native genera of submerged macrophytes (and charophytes) used for revegetation in wetlands; (**b**) the most used native species of submerged macrophytes for revegetation in wetlands. (**c**) Distribution of the native genera of floating-leaved and free-floating species used for revegetation in wetlands. (**d**) Percentage of the floating-leaved and free-floating native species used for revegetation in wetlands.

Among the criteria to select the hydrophyte species for revegetation is the availability of knowledge on each species. For example, methods for collecting, processing and storing large quantities of *Ruppia maritima* and *Potamogeton perfoliatus* seeds started in 2004 and protocols for using seeds of these species in restoration plantings are described in Ailstock et al. [66]. *Myriophyllum spicatum,* a perennial submerged macrophyte, is one of the species preferentially used in many restoration projects in lakes and wetlands [103] (see Table 1 and Table 2 and Figure 4), mainly due to its strong resistance to pollution. Because of environmental disturbances, *M. spicatum* is easily broken to form apical fragments and then it is possible for them to develop into robust new plants and gradually settle to form colonies. *M. spicatum* can also tolerate both fresh and brackish water [104,105]. This tolerance range allows *M. spicatum* to live under a wide range of salinity and different oxidative stress conditions. This makes this species a good candidate for coastal wetlands affected by salinization [106]. Moreover, *M. spicatum* can secrete allelochemicals to inhibit the growth of microalgae [107]; the major components of these secondary metabolites secreted by plants are phenolic acids, fatty acids, alkaloids, terpenoids, flavonoids, etc. [108]. Other species which produce and secrete allelopathic compounds are, for example, *Vallisneria spiralis* [109], *Ceratophyllum demersum* [110], *Potamogeton malaianus* [111], and also charophyte species [112,113]. The allelopathy of macrophytes on microalgae growth is extremely promising due to its low cost, good algal inhibition effect and high environmental safety [114] and should be also a criterion to consider in the selection of hydrophytes for revegetation. The use of allelochemicals produced by macrophytes in the field of water ecological restoration has been recently reviewed by Li et al. [108]. These authors even propose searching in the micro-spheroidization technology as engineering applications of allelochemicals directly in water to prevent microalgal growth.

Regarding flow-surface constructed wetlands [115], *Ceratophyllum demersum*, *Hydrilla verticillata*, *Myriophyllum verticillatum*, *Vallisneria natans*, and *Potamogeton crispus* are commonly used among the submerged plants. The commonly used free-floating hydrophytes in CWs include *Lemna minor*, *Eichhornia crassipes*, *Salvinia natans*, and *Hyrocharis dubia*. Meanwhile, floating-leaved species in CWs are mainly *Nymphoides peltata*, *Trapa bispinosa*, *Nymphaea tetragona*, and *Marsilea quadrifolia* [116].

The use of resistant genotypes (to herbivores and salinity, for example) in hydrophyte restoration, as it is proposed for seagrasses [117], might be an approach for improving the extant genetic baselines of natural populations and for enhancing the resilience of the restored population to present and future stressors (e.g., climate change). The selection of more tolerant genotypes to improve restoration success could be performed by growing wild specimens under controlled conditions, but resistant genotypes can also be produced with a lower level of intervention through the use of priming/hardening methods [118]. Pre-exposing specimens to mild stress has the potential to induce stress memory, giving rise to genotypes with enhanced tolerance to subsequent stressful events [118]. When stress memory is set by stress-induced epigenetic modifications, the acquired resistance can be passed to offspring leading to new generations with acquired resistance. Therefore, when dealing with clonal plants, such as most submerged and floating hydrophytes, restoration management should consider “epigenetic diversity” as an indicator of stability and functioning of the ecosystem equal to genetic diversity. However, this is a still unexplored issue in the field of wetlands restoration with hydrophytes.

## 7. Factors Affecting the Success of Restoration

### 7.1. Site Selection

Site selection is arguably one of the most critical steps in wetland restoration processes [44]. Many restoration projects have failed due to inadequate site selection. Among the factors to be considered are the historical presence or absence of submerged aquatic vegetation, water depth and light availability, water column nutrient concentrations, sediment quality, wave exposure, etc. Interannual variability in climate and water quality conditions (see below) also play a critical role in the initial establishment and survival of planted submerged aquatic vegetation. This is why planting efforts may need to be repeated over multiple years to achieve great success [44]. Regarding climate variability, site selection should consider the foreseen changes that will occur due to climate change in the near future that will surely affect the success of revegetation [119].

### 7.2. Time Selection

Introduction of hydrophytes should be carried out early in the favorable season in eutrophic wetlands, before the development of microalgae and/or filamentous algae (see below). However, the period for plant introduction should not affect other important phenological events such as the breeding of waterfowl [120]. Rodrigo and Segura [121] reported unsuccessful revegetation in 2020, due to an inappropriate time for it in a Mediterranean wetland. The revegetation was planned for mid-March 2020, and all cultures were prepared, but then the lockdown of the whole society was declared due to the pandemic caused by the SARS-CoV-2 virus. The planting was finally performed in mid-June 2020, when the “normal” people mobility situation was restored. However, the hydrophyte recovery failed due to the intensive growth of filamentous green algae, which had already developed at this time of the year; hydrophytes could not outcompete with the filamentous algae. In the planning for planting, the acclimation time of the hydrophytes in the field has to be also considered.

### 7.3. Herbivory

Herbivory can be performed mainly by waterfowl, fish, crayfish and turtles. Moore et al. [49] pointed at herbivory as the main factor for the lack of success in the restoration with *Vallisneria americana*. The growth of both adult transplants and plants developing from seeds was good, but using mesh exclosures to protect the plants from herbivory proved to be critical to the restoration success. Similar results were obtained by Rodrigo et al. [16] in Mediterranean wetlands. Studies in the United Kingdom [122,123], Denmark [46] and Germany [47] showed higher survival and number of plants and longer total shoot length in enclosures that prevented bird access. Thus, long-term protection with exclosures may be required to establish large founder colonies that are of sufficient size to withstand initial grazing pressures (Figure 3b). The size of the exclosures can be progressively enlarged to obtain wider surface coverages each time [86] (Figure 3c). However, the use of such protective exclosures as a restoration tool for very large-scale use is difficult, due to high costs for material, installation and maintenance and difficulties, such as filamentous algal growth (see below) and interference with fauna and recreational use.

Herbivore species may have preferences on particular species. For example, Yu et al. [124] described how grass carp preferred *Vallisneria spinulosa* and *Ceratophyllum demersum* to *Myriophyllum spicatum*. Waterfowl have been documented to graze selectively on *Stuckenia pectinata* (herbivores electively removed *S. pectinata* specimens in favor of charophytes in Estonia [125], and waterfowl suppressed dominance of *S. pectinata* in favor of subordinate *Zannichellia palustris* and *Potamogeton pusillus* in The Netherlands [126]). Invasive red swamp crayfish preferentially fed on charophytes [127]. Therefore, having a good knowledge of the herbivores present in the wetland is also essential for planning the species selection. Finally, to determine the likelihood of consumption and resistance to disturbances of the different macrophyte species, both hydrophyte palatability and disturbance tests should be carried out directly in the field. 

### 7.4. Massive Filamentous Algal Development

Since the wetlands to be restored are, in most cases, eutrophic systems, submerged macrophyte recovery is often accompanied by an excessive proliferation of filamentous green algae [128]. Filamentous algae compete with submerged macrophytes for space, light, nutrients and other resources; they also mechanically damage hydrophyte stems and leaves by twining around them, negatively influencing their normal growth. Moreover, the response of regeneration ability of apical fragments to decaying green filamentous algae is negatively affected (see, for example, the adverse influence of *Cladophora oligoclona* on *Hydrilla verticillata* seed germination and seedling growth [129] or on *Myriophyllum spicatum* formation of buds and roots [128]. Furthermore, high growth rates, as high as 0.7–0.8 d^−1^ [130], have been described for several species of *Cladophora* and also can grow from their internal nutrient storages. This confers a large advantage over hydrophytes. Since filamentous green algae grow adhered to substrates in their benthic stage [131], planted hydrophytes (as well as the nets of the exclosures, see above), can be used by them as suitable substrates. All this can lead to the recession or even the disappearance of the hydrophytes in the restored system. Zhang et al. [132] suggested the importance of appropriately selecting macrophyte species to prevent filamentous algal bloom in shallow water bodies restoration. They recommended avoiding planting of *H. verticillata* and *C. oryzetorum* because these species promoted the growth of filamentous algae in the early spring, while *P. malaianus* might inhibit filamentous algae and this species was recommended as a pioneer species. Therefore, the excessive growth of filamentous green algae should be regulated during revegetation, although this is a real challenge in the restoration of eutrophic wetlands. Bearing climate change in mind [133], the increase of temperature foreseen for areas such as the Mediterranean would favor the early development of green filamentous algae in wetlands located in that region. The removal of the dense mats formed by the filamentous algae and the use of this waste in collaboration with biotechnological companies could be a solution, since the use, for example, of *Cladophora glomerata* removed from sites where it forms green tides [134] is described for the production of highly crystalline cellulose [135]. In this way, similar to what is done with the withdrawal of emergent vegetation biomass in constructed wetlands, three goals can be achieved: (i) elimination of large quantities of nutrients from water which are now retained in the filamentous algal biomass, (ii) the use of waste which represents an important environmental problem, and (iii) help the revegetation with hydrophytes.

### 7.5. Water and Sediment Quality Conditions

Wetlands to be restored are frequently rich in water and sediment nutrients but also in contaminants, such as metals or organic compounds. These concentrations should be reduced below the tolerance thresholds of the hydrophyte species prior to being reintroduced. Experimental mesocosm studies performed in Denmark indicated that the threshold concentrations above which is likely to lose submerged macrophytes in shallow systems are 1.2–2 mg/L of TN and 0.13–0.20 mg/L of TP [136,137]. Wang et al. [138] found the TP thresholds for the shift from clear-water state to turbid-water state at 0.08–0.12 mg/L. Submerged macrophytes cannot tolerate high ammonia concentrations. This compound may cause damage to and loss of macrophytes in wetlands and shallow lakes [139]. The threshold value of ammonia for *Potagometon crispus* has been stated as 4 mg/L [140], but it is said that ammonia tolerance differs greatly among wetland plant species [141]. Wang et al. [142] found that the increase of TN removal efficiency in *Myriophyllum aquaticum* was hindered when treated with high NH_4_^+^ concentration (26–36 mmol/L), suggesting this as the threshold for its tolerance to NH_4_^+^. Regarding nitrogen and charophytes [143,144], Lambert et al. [144] predicted a transition from charophyte presence to absence in aquatic ecosystems at a concentration of approximately 2 mg NO_3_-N/L. However, Rodrigo et al. [102] found *C. hispida* and *C. vulgaris* forming meadows with nitrate concentrations higher than 2 mg NO_3_-N/L in water bodies affected by seepage from agricultural runoff. Moreover, in laboratory experiments, these species grew well up to 30 NO_3_-N/L. Performed research related to the use of submerged macrophytes in constructed wetlands has provided a wide knowledge in terms of thresholds for water quality conditions for particular species (e.g., [141,142]). Some wetland hydrophytes are being used as phytoremediating plants capable of taking up heavy metals and other pollutants from water and sediments. For example, *Ceratophyllum demersum* can remove cadmium from sediments by phytoextraction by means of the production of phytochelatin for metal binding in shoots [145,146], *Potamogeton pectinatus* and *P. malaianus* has also been attributed a high capability to remove heavy metals and other pollutants directly from the contaminated water [147,148]. Among charophytes, *Chara vulgaris* has been lately proposed to be used in phytoremediation [149,150,151]. As the tolerance to nutrients and different pollutants varies among the hydrophyte species, this is also an important aspect to take into account when selecting plants species for wetland restoration according to the state of water and sediments in each particular wetland.

## 8. Evaluation of the Success of Revegetation

Revegetation should be evaluated at different scales in both spatial (from the community up to the landscape) and temporal (from seasonal dynamics up to long-term changes) dimensions [44]. This requires approaches that are, at the same time, effective and feasible in the long-term. Some revegetation projects have been followed in the long-term [92], however, others were leaved behind at relatively early stages due to the huge undertaking a meaningful follow-up represents a, and/or because scientists frequently lack funding and the necessary opportunities, and, finally, because of the lack of interest of developers [92]. 

The monitoring can be done by using control plots and aerial photography surveys and other remote sensing methods, when possible. Unmanned (or Unoccupied) Aerial Vehicles (UAVs), known by the popularized name of drones, have been utilized for algal bloom and submerged aquatic vegetation detection for nearly two decades [152]. This type of high-resolution aerial imagery offers a cost-effective and rapid method to assess primary producer assemblages in aquatic environments, and provides great spatial resolutions for imaging [153]. Moreover, UAVs have advantages over manned vehicles for remote sensing: (i) flying UAVs is less expensive, (ii) is more flexible in scheduling, (iii) enables lower altitudes, (iv) uses lower speeds, (v) and the already cited provision of better image spatial resolution. Mistch et al. [154] used color aerial photography followed by ground-truth verifications (normalized maps and a grid system marked with permanent, numbered white poles to facilitate identification of the locations of plant communities in the wetland during ground-truthing and aerial photography). However, permissions are required in many countries for the use of UAVs [155]. Reflectance and transmittance spectra of floating-leaved plants can be measured, to know their influence on light availability in the water column which can alter the environmental conditions underneath the water surface [156]. With the data obtained at the ground level, macrophyte community diversity indexes should be applied to examine if the desired goals in terms of hydrophyte biodiversity have been achieved (see for example [154]).

## 9. Final Remarks and Conclusions

Restoration of wetlands by revegetation with native hydrophytes is a challenging task. Several countries have developed pilot case studies and field-scale projects with more or less success. The number of large field-scale cases are less due to all the needed issues that have to be solved (not only biological but financial, staff resources, etc.). Most published papers (more than 90%) only refer to successful results, but study cases in which failure in revegetation has been the outcome and that analyze the reasons for such a result, should be published as well, to learn from “what not to do”. Some of the shortcomings of experimental designs which could significantly limit the interpretation of hydrophyte reintroduction projects are: (i) inadequate previous information and documentation, (ii) lack of understanding of the underlying reasons for the decline in existing plant populations, (iii) poorly defined success criteria for revegetation projects, (iv) insufficient monitoring following reintroduction, which can drive to an overly optimistic evaluation of success based on short-term results. Clearly, successful revegetation needs to be accompanied (in advance) by other management actions, such as external and internal nutrient load reduction, food web biomanipulation, increasing light availability by water level drawdowns in spring, etc. (for detailed information see Hilt et al. [47]). Moreover, before starting revegetation, the existence of any legal restrictions should be checked, because they can be different in each country.

It can be concluded that the value of hydrophyte revegetation as a restoration tool could be improved by:

(i) Performing research in advance. Experimental out-site (culture room) and on-site (wetland mesocosms) should be planned before starting larger-scale initiatives.
(ii) Selecting suitable wetlands with ecologically suitable revegetation sites. It is very important to consider the clear negative factors which prevent the success of revegetation (herbivory, microalgae and filamentous green algae, etc.). If revegetation is performed in sites with high nutrient and pollutant concentrations, high density of herbivorous fish, very low water transparency, etc., the result will be a total failure [47].
(iii) An increased focus on species biology (including genetics) and ecology. Selecting and obtaining native (and typically occurring in the wetland previous to its degradation) suitable hydrophyte species is fundamental. In the studies reviewed here, the use of floating hydrophyte species has been less generalized than the submerged species. A total of 45 different species of submerged hydrophytes and 14 floating-leaved and free-floating species have been used for revegetation in wetlands (Figure 4). The genus *Potamogeton* has been used the most among the submerged hydrophytes (in 29% of the occasions), but *Myriophyllum spicatum* and *Hydrilla verticilata* have been the two most used species (15% and 13%). The genus *Nymphaea* has been the most used as a free-floating hydrophyte (36% of occasions), followed by the floating-leaved species of *Potamogeton* (22%). Introducing highly competitive species (r-strategists) has the risk that they outcompete part of the original vegetation including rare species. However, if the initial aim is to have a large cover of hydrophytes to prevent the growth of phytoplankton, resuspension of the sediment, etc., they can be chosen, and, in a second step, other species, specifically rare species, could be reintroduced in particular sites suitable for them. Although other management actions had been applied (i.e., nutrient and pollutant reductions), species or ecotypes/genotypes with high capacity to tolerate stress conditions should be initially chosen. *Potamogeton pectinatus*, *P. malaianus*, and *Ceratophyllum demersum* can live in contaminated water with heavy metals and other pollutants and remove them [145,146,147,148]. Among charophytes, *Chara vulgaris* is maybe the best candidate [102,149,150,151]. The selection of species with high allelopathic capacity against phytoplankton and periphyton is a complementary issue (e.g., *Myriophyllum spicatum* [107], *Vallisneria spiralis* [109], *Ceratophyllum demersum* [110], *Potamogeton malaianus* [111]). *P. malaianus* also inhibits filamentous algae growth.
(iv) Deciding the appropriate wetland surface area to be potentially planted with hydrophytes. To increase light availability and be sure that clear-water conditions will be maintained, this area should be at least 30–40% of the wetland surface where hydrophytes could grow (this has to be determined in advance, based on wetland morphometry, water column light attenuation, light requirements for growth of the selected species according to their type, such as caulescent or rosette-type angiosperms, charophytes, etc.).
(v) Selecting the appropriated revegetation techniques, considering the seed production and recruitment. The studies reviewed here suggest that sediment transfer is more adequate for temporary wetlands. However, in the cases of transferences from other sites to the target wetland, nature protection aspects and the potential risk of transferring pollutants, fish parasites, pathogens or other undesired species must be considered. Samples of this sediment have to be chemically analyzed to dismiss the presence of different kinds of pollutants and also carefully observed by experts to be sure that no unwanted propagules are present. If nutrient or pollutant contents are high, experimental tests of the sediment suitability by planting test species are recommended.
(vi) Choosing the suitable propagation technique. The advantages of seed-based approaches, in comparison to other revegetation techniques when large areas are considered, are the lower cost and the better logistical feasibility [1]. For seeding, densities varying from 11 to 100 seeds/m^2^ have been used for coastal wetlands. A high number of “transplants” and of adequate length should be selected: around 10 ramets/m^2^ with lengths of 20–30 cm seem to be the most adequate to be planted (with apical parts) [7,17,46]. The use (or not) of a substrate to plant the prepared cultures in the wetland will depend on the type of the radicular system the hydrophyte develops and the features of the receptor sediment. Hydrophytes, such as *M. spicatum*, *S. pectinata* or *C. vulgaris,* for example, do not need any kind of support substrate. If the sediment is unconsolidated with low cohesive strength—typical for waterbodies with previous phytoplankton dominance—degradable substrates should be used. Planting by hand, although work-intensive, can be achieved by involving volunteers. Mechanical planters might have a great impact on the wetland fauna. When a moderate herbivory pressure on hydrophytes is suspected, protective exclosures should be used in initial trials to determine if the magnitude of this pressure will cause the failure of the revegetation. Protective exclosures can be also used, progressively enlarging them until established hydrophyte stands resistant to herbivory are formed to facilitate submerged macrophyte growth and dispersal.
(vii) Performing long-term monitoring programs to assess the performance and the variability of the restored populations over time. Whole-ecosystem, long-term interventions including most if not all ecosystem processes are desirable to be sure that the restoration result is the expected [62]. Furthermore, for large-scale hydrophyte restoration, the efforts should be in the framework of coordinated interagency programs, to develop, evaluate, and refine the suitable protocols and procedures. All this information will allow modeling the transition to an alternative stable clear macrophyte-dominated state and its future resilience [157].

It is necessary to encourage countries to publish scientifically sound guidelines to help managers aiming to restore wetlands and shallow lakes, and critically assess and predict the potential development of submerged vegetation, taking into account the complex factors and interrelations that determine their occurrence, abundance and diversity. Despite all the information already found in the published documents regarding revegetation with hydrophytes (approaches and experiments, manipulations in the field, etc.), further research is needed to key issues, such as target recruitment bottlenecks, interactive factors, foreseen climate change, etc., specific to many species and wetland types, which can shed light about how to maximize the recruitment and ensure restoration, for example, regarding the selection of species or the types of environmental manipulations [1]. Not only ecology but also microbiology, soil and genetic sciences are necessary to improve the success of revegetation with hydrophytes, because they can provide new insights into why revegetation fails. The inclusion of an “epigenetic restoration and conservation” perspective together with a genetic one is also desirable as has been suggested for seagrass restoration [117]. Many papers lack precise data on the speed and efficiency of colonization of the wetlands by the different species, and this information is very valuable for wetland restoration practices with hydrophytes elsewhere.

Finally, the revegetation with hydrophytes must be performed in the context of broader wetland habitat restoration projects to have a greater chance of success. Restoration needs a continued effort (in terms of time and economic and personal resources) of research and implementation. It is clear that research so far has been very productive, but the results obtained should be more effectively integrated with policy-making, general wetland restoration practices and with a landscape perspective [158], particularly under future climatic scenarios.

## Figures and Tables

**Table 1:** Summary of outdoor experiments performed to plan larger-scale revegetation with hydrophytes. Plant species, some experiment features, and site (country) are indicated. References are ordered chronologically. N/C indicates if the wetland/shallow lake is natural (N) or constructed (C).

Planted Hydrophyte Species		Plant Origin	Experiment Features	Site (Country)	N/C	Ref.
Submerged	Floating-Leaved					
*Vallisneria* sp. *Hydrilla verticillata* *Potamogeton maackianus*	*Trapa bicornis* *Nelumbo nucifera*	Not indicated	800–3000 m^2^ enclosures in three sublakes	The shallow lake Donghu (China)	N	[45]
*Stuckenia pectinata* *Potamogeton perfoliatus* *P. lucens*	--	Collected 80 km south of the lake in ditches and channels	25 m^2^ protected and unprotected areas	The shallow lake Engelsholm (Denmark)	N	[46]
*Myriophyllum spicatum* *Chara contraria*	--	Not indicated	200 m^2^ “macrophyte islands”	A shallow lake (Germany)	N	Rott (2005) in [47]
*Hydrilla verticillata, Potamogeton malaianus* *Vallisneria spiralis* *Najas marina*	--	Wuli Bay and East Taihu Bay (lake Taihu)	200 L containers in an outdoor green house	The shallow lake Taihu (China)	N	[48]
*Vallisneria americana*	--	From nursery grown stock in culture ponds at Virginia Inst. Marine Sci. campus; seed pods from beds in Nanjemoy Creek, Maryland; separated seeds	4 exclosures of 40 m^2^ (with 2 × 2 m plots inside)	A tidal marsh area at James River (VA, USA)	N	[49]
*M. spicatum* *S. pectinata* *Chara hispida* *Nitella hyalina*	--	From cultures produced in indoors culture room	54 exclosures of 1 × 1 m	Tancat de la Pipa wetland (Spain)	C	[16]
*P. malaianus* *M. spicatum* *H. verticillata* *V. spinulosa*	--	From lake Taihu and cultivated in outdoor tanks	100 outdoor tanks (680 L; 100 cm diam. 100 cm height)	Gonghu bay, lake Taihu (China)	N	[50]
*Elodea canadensis Myriophyllum alterniflorum Ceratophyllum demersum*	*Potamogeton natans* (but appear spontaneously)	Shoot fragments from nearby ponds	6 surface-flow constructed semi-natural wetlands (10 × 4 m)	Semi-natural wetlands in agricultural landscape (Sweden)	C	[51]
*Heteranthera* *dubia*	*Potamogeton* *nodosus*	Founder colonies from nearby sites	24 ring cages (0.9-m diam.)	Dallas Floodway Extension Lower Chain of Wetlands (USA)	C	[52]

**Table 2:** Summary of some larger (>0.4 ha) field revegetation with hydrophytes. Plant species used, surface treated, and site and country are indicated. References are ordered chronologically.

Planted Hydrophyte Species		Surface	Site (Country)	Reference
Submerged	Floating-Leaved/Free Floating	(ha)		
*Chara australis*	--	1	Shallow lake Rotoroa (NZ)	[55]
*Ceratophyllum demersum* *Myriophyllum verticillatum* *Myriophyllum spicatum*	*Nymphaea alba*	1.5 and 1.2	Almenara and Algemesí wetlands (Spain)	[56]
*Potamogeton malaianus* *Myriophyllum spicatum* *Potamogeton maackianus* *Hydrilla verticillata* *Vallisneria natans*	*Nymphoides peltata* *Nymphaea rubra* Trapa bicornis *non-native *Alternanthera philoxeroides*	10	Large enclosure in Lake Wuli, northern bay of Lake Taihu (China)	[57]
*Potamogeton cheesemanii* *Myriophyllum propinquum*	*Lemna minor*	--	Several wetlands in New Zealand	[40]
*Myriophyllum spicatum* *Stuckenia pectinata* *Ceratophyllum submersum*	--	6 and 8	Educative and Reserve lagoons, Tancat de la Pipa wetland (Spain)	Sebastián and Peña in [16]
*Ceratophyllum demersum* *Chara vulgaris* *Heteranthera dubia* *Potamogeton illinoensis* *Potamogeton pusillus* *Vallisneria americana* *Zannichellia palustris*	*Potamogeton nodosus Nelumbo lutea* *Nymphaea mexicana* *Nymphaea odorata*	>10	Chain of wetlands at Dallas Floodway Extension (USA)	[58]
*Hydrilla verticillata* *Vallisneria spinulosa* *Potamogeton maackianus* *P. malaianus* *M. spicatum* *Ceratophyllum demersum*	--	5, 8 and 0.4	Shallow lakes Wuli (isolated bays of lake Taihu), Qinhu and South (China)	[59]
*Vallisneria americana*	*Potamogeton nodosus/natans* *Nymphaea odorota*	~18	Great Lakes wetland area (Canada)	[60]
*P. malaianus* *M. spicatum* *H. verticillata* *V. spinulosa*	--	0.4	Gonghu Bay, Lake Taihu (China)	[36]
*Vallisneria denseserrulata Hydrilla verticillata*	--	12	One basin of Huizhou West shallow lake (China)	[61]

**Table 3:** Some examples of practices with wetland sediment/soil transfers. References are ordered chronologically.

Recovered Species	Sediment Origin	Receptor	Site (Country)	Reference
*Chara braunii, Nitella hyalina, Monochoria korsakowii, Nymphoides peltata, Limnophila sessiliflora, Vallisneria denseserrulata, Hydrilla vercillata*, *Ceratophyllum demersum* and five species of *Potamogeton*	Seed banks from lake-bottom sediments	Lake shores ranging 5300–27,800 m^2^ (width: 30–60 m). Sediments spread thinly (~10 cm)	Littoral areas of shallow Lake Kasumigaura (Japan)	[91,94]
(Mostly emergent plants) *Myriophyllum spicatum*	0–5 cm deep soil from 1 × 1 plots from different sites	Surface of 55 m^2^	Yeyahu wetland natural reserve (China)	[90]
*Callitriche* sp., *Callitriche truncata*, *Chara aspera, C. canescens, C. globularis, Ranunculus peltatus, R. trichophyllus, Tolypella glomerata, T. hispanica, Zannichellia obtusifolia, Z. pedicellata*	40 L (from 45 × 45 cm, 3 cm deep) of soil per donor site (5 temporary wetlands)	50 L of soil on a 4 × 2 m plots at the bottom of each transfer mesocosm	Cassaïre site, Camargue area (France)	[92]
