# Correction: Rodrigo, M.A. Wetland Restoration with Hydrophytes: A Review. *Plants* 2021, *10*, 1035

**DOI:** 10.3390/plants14203161

**Published:** 2025-10-15

**Authors:** Maria A. Rodrigo

**Affiliations:** Integrative Ecology Group, Cavanilles Institute for Biodiversity and Evolutionary Biology, University of Valencia, Catedrático José Beltrán 2, 46980 Paterna, Valencia, Spain; maria.a.rodrigo@uv.es

In the original publication [1], the work by Shutes [2] was not cited. The citation has now been inserted in the first paragraph of Section 2 and should read as follows: Surface-flow CWs are similar to natural marshes, usually located in shallow channels and basins through which the water flows slowly above the substrate [20].

Reference [3] was not cited. The citation has now been inserted in the fourth paragraph of Section 5.1 and should read as follows: To vegetate large wetland areas, aerial seeding might be both economical and practically feasible [77]. With this, the restoration efforts could be accelerated without using expensive hand-planting methods of vegetative clones (see below), but the unavailability of large quantities of viable seed is one of the major hindrances [77].

Some wording changes have been made throughout the text in Sections 1, 2, 4, 5, 7 and 8, and some repetitions of citations have been added (e.g., [1]: Kettenring, K.M.; Tarsa, E.E. Need to Seed? Ecological, Genetic, and Evolutionary Keys to Seed-Based Wetland Restoration. *Front. Environ. Sci*. **2020**, *8*, 109, and [9]: Orsenigo, S. Editorial: How to halt the extinction of wetland-dependent plant species? The role of translocations and restoration ecology. Aquat. Conserv. *Mar. Freshw. Ecosyst.* **2018**, *28*, 772–775).

With these corrections, the order of some references has been adjusted accordingly. 

The authors state that the scientific conclusions are unaffected. This correction was approved by the Academic Editor. The original publication has also been updated.
